# Understanding of Medication Information in Primary Health Care: A Cross-Sectional Study in a South Eastern European Population

**DOI:** 10.3389/fpubh.2020.00388

**Published:** 2020-08-11

**Authors:** Dajana Roshi, Genc Burazeri, Peter Schröder-Bäck, Ervin Toçi, Salvatore Italia, Alban Ylli, Helmut Brand

**Affiliations:** ^1^Department of International Health, School CAPHRI (Care and Public Health Research Institute), Maastricht University, Maastricht, Netherlands; ^2^National Agency for Drugs and Medical Devices, Tirana, Albania; ^3^Department of Public Health, Faculty of Medicine, University of Medicine, Tirana, Albania

**Keywords:** Albania, family physicians, information, medication use, primary health care, socio-demographic factors

## Abstract

**Aim:** We aimed to assess adult primary health care (PHC) users' understanding of their medication information in a transitional South Eastern European population across seven domains.

**Methods:** A cross-sectional study, carried out in Albania in 2018–19, included a representative sample of 1,553 PHC users aged ≥18 years (55% women; overall mean age: 54.6 ± 16.4 years; overall response rate: 94%). Participants were asked about their understanding of information they received from their respective family physicians about prescribed medicines in terms of factors like cost, dosage, and side-effects. Socio-demographic data were also gathered. Binary logistic regression was employed to assess the socio-demographic predictors of information about medication use and administration.

**Results:** Across different aspects of use and administration, 21–60% of participants did not understand their medications. Less understanding of medication use was particularly high among the poor and those with low education and among urban residents, irrespective of socioeconomic status.

**Conclusion:** This study provides important evidence about the level and socio-demographic determinants on understanding of information about medication use and administration among adult PHC users in a transitional former communist country in South Eastern Europe. Policymakers should be aware of the joint role and interplay between health literacy (demand side) and information provision (supply side), which both significantly influence the understanding of medication use by the general population.

## Introduction

According to the World Health Organization (WHO), the rational use of medicines is defined as follows: “*Patients receive medications appropriate to their clinical needs, in doses that meet their own individual requirements, for an adequate period of time, and at the lowest cost to them and their community*” (1). Subsequently, WHO published a list of 12 core components that promote rational use of medicines, which include governmental and other sectoral and inter-sectoral interventions toward this aim targeting simultaneously the whole system, the supply (health care providers) and demand (health care consumers) side (2). Such interventions range from coordination of medicine use policies; clinical guidelines and essential medicines lists; pharmacotherapy training in undergraduate curricula; supervision, audit, and feedback; independent information on medicines to public education about medicines; avoidance of inappropriate financial incentives; as well as other effective measures (2).

In order to adequately and safely prescribe medicines, health personnel must effectively explain medication use and administration to patients, a process that is increasingly complex given the increasing number and heterogeneity of medicines available (3). To this end, the intertwined and multidirectional approaches and interventions contained in the WHO's list of 12 core components might play a crucial role toward the enhancement of health personnel knowledge about medicines, a process that could improve both the use of medicines and the way patients are informed.

Indeed, the way medical information is conveyed to consumers might also influence patients' understanding of the medical information and adherence to treatment regimens, with written materials valued most by primary health care consumers and pharmacists and spoken information ranked as the preferred method for the provision of medical information by general practitioners (GPs) (4). For GPs, the main barrier to provide written medicines information to patients was the limited time in disposal or being too busy. Among GPs, the prevailing of spoken information to patients is mainly driven by the lack of time together with physicians' concerns about patients' capacity to understand written medicine information (4), with the latter being entailed in the broader concept of health literacy (HL).

In general, the HL of health care consumers or the ability to obtain, process, understand, and apply health information into appropriate health decisions (5), including proper medication use and administration, might be influenced by various demographic and socioeconomic factors such as age, education, and occupation (5, 6). Inadequate HL might be associated with impaired ability to understand medication and treatment information provided by physicians, lower participation in the care process, being less prone to ask questions about medical care issues (7, 8) and impaired ability to follow medical instructions (9).

The evidence about medication use and medication information in primary health care is limited for Albania, a country with low health expenditure but relatively good health indicators (10), struggling to join the European Union (11). After the breakdown of the communist regime in 1990, Albania has experienced profound political and socioeconomic reforms which have been also associated with changes in the epidemiological profile and health characteristics of the general population (12). Life expectancy in Albania in 2018 was 77.4 years in men and 80.5 years in women (13). The burden of non-communicable diseases (NCDs) has increased about 45% during the period 1990–2017 (14). Since 2018, a reform of governance of primary and secondary health care system has been underway in Albania. A new central institution referred to as the “General Operator of Health Care Services” with four regional branches (“Regional Operators”) has already taken over some responsibilities from the Ministry of Health and Social Protection in planning and management of public health programs; primary health care centers and regional/municipal hospitals.

In this framework, the aim of this study was to assess adult primary health care (PHC) users' understanding of their medication information across seven domains in the general population of Albania, a transitional former communist country in South Eastern Europe. We hypothesized a lower level of understanding of the information about medication use among: the low socioeconomic groupings (the poor and the least educated individuals); and, irrespective of the socioeconomic status, among urban residents (due to the tremendous workload and time-pressure of Albanian physicians operating in the cities).

## Materials and Methods

A cross-sectional study was carried out in Albania during December 2018–January 2019.

### Study Population and Sampling

Study population involved adult PHC users in five major regions of Albania (Tirana, Shkoder, Vlore, Fier, and Diber). WINPEPI, a freeware package of statistical programs for epidemiologists (15), was employed for sample size calculation. The minimal required sample size was conservatively calculated at about 870 participants. Nonetheless, we planned to recruit 1,500 adult individuals, of whom 500 in Tirana region and 250 individuals in each of the other four regions of Albania. In Tirana, two PHC centers (with probability proportional to size, PPS) were selected in urban areas and other two PHC centers were selected in rural areas (with PPS). In addition, in each of the other four regions, one PHC center (with PPS) was selected in urban areas and another PHC center (with PPS) was selected in rural areas. Subsequently, in each of the 12 selected PHC centers (four centers in Tirana and two centers in each of the other four regions), a consecutive sample of male and female PHC users aged ≥18 years was included in the study until reaching the expected quota [500 individuals in Tirana region (250 in urban areas and 250 in rural areas) and 250 individuals in each of the other four regions of Albania (in each region: 125 individuals in urban areas and 125 individuals in rural areas)].

On the whole, during the period December 2018–January 2019, there were 1,649 individuals aged 18 years and above who attended the 12 selected PHC centers in the five study regions. Of these, 39 individuals could not be interviewed due to their advanced stage of diseases, whereas other 57 individuals refused to participate. Thus, 1,553 individuals were included in this study, with a response rate of: 1,553/1,649 = 94.2%.

### Data Collection

A structured questionnaire was administered by trained interviewers to all individuals who agreed to participate in this study. Participants were asked about their understanding of information they received from their respective family physicians about prescribed medicines in terms of factors like cost, dosage, and side-effects. More specifically, participants were asked the following questions:

In general, when prescribed medicines by your family physician:

- Do you understand the *names* of medications prescribed?
- Do you understand their *dosages*?
- Do you understand the *reasons* for medications' prescription?
- Do you understand the *hours* of medications' intake?
- Do you understand the *duration* of medications' intake?
- Do you understand the *side effects* of the treatment prescribed?
- Do you understand the information about the *cost* of treatment?

Possible answers to each question were: yes vs. no.

In addition, data about socio-demographic factors of study participants was gathered, including age (<40, 40–64.9, and ≥65 years), sex, marital status (married vs. single, cohabiting, divorced, or widowed), place of residence (urban vs. rural areas), education [low [0–8 years of formal schooling], middle [9–12 years], high [≥13 years of formal schooling]], employment (employed, unemployed, retired) and self-perceived poverty level (poor vs. not poor).

Beforehand, the questionnaire was successfully pretested in urban and rural PHC centers in Tirana in an overall sample of 56 adult PHC users (37 females and 19 males; overall median age: 58 years). The pretest findings indicated that 46–82% of males and females interviewed usually understood the information about different aspects related to the use of medications prescribed by their family physicians.

The study was approved by the Albanian Committee of Bio-Medical Ethics in November 2018. All participants included in this study provided their consent after being informed in detail about the objectives and procedures of the study.

### Data Analysis

Fisher's exact test was used to compare the distribution of information about medication use and administration (seven separate outcome variables, each dichotomized into: yes vs. no) between male and female PHC users included in this study.

Conversely, binary logistic regression was used to assess the association between information about medication use (seven outcome variables) and socio-demographic characteristics of study participants (predictors). Firstly, crude (unadjusted) odds ratios (ORs: not understood vs. understood, for each of the seven outcome variables), their respective 95% confidence intervals (95% CIs) and *p*-values were calculated. Secondly, all demographic factors and socioeconomic characteristics (age, sex, marital status, residence, education, employment, and poverty) were entered into the logistic regression models and removed in a backward stepwise procedure if their *p*-value exceeded 0.10. Multivariable-adjusted ORs, their respective 95% CIs and *p*-values were calculated. The Hosmer-Lemeshow test was used to assess goodness-of-fit; all analyses fitted the criterion.

A *p*-value of ≤0.05 was considered as statistically significant for all statistical tests conducted. All statistical analyses were done with Statistical Package for Social Sciences (SPSS, version 19.0).

## Results

Of 1,553 participants included in this study, 16 (0.4%) individuals had never been prescribed medicines by their respective family physicians. On the whole, 1,530–1,537 individuals (98–99% of the overall study sample) provided valid data on different aspects related to the information about use and administration of medications prescribed by their family physicians. The overall mean age in this study sample was 54.6 ± 16.4 years.

Table 1 presents the information about medication use provided by family physicians to study participants. Across different aspects of use and administration, 40–79% of participants understood their medications. Hence, about 73% of participants usually understood the *names* of medications prescribed; 71% usually understood medications' *dosages*; 68% usually understood the *reasons* for medications' prescription; 79% usually understood the *hours* of mediations' intake; 77% usually understood the *duration* of medications' intake; 66% usually understood the *side effects* of the treatment; however, only 40% usually understood the *cost* of the treatment.

**Table 1:** Information about medication use provided by family physicians to a representative sample of primary health care users in Albania in 2018–19.

**Outcome variable**	**Total**	**Men**	**Women**	***P*^**b**^**
**Usually, understood the medications' names:**				
Yes	1,113 (72.6)^**a**^	507 (72.9)	606 (72.2)	0.774
No	421 (27.4)	188 (27.1)	233 (27.8)	
*Total*	*1,534 (100.0)*	*695 (100.0)*	*839 (100.0)*	
**Usually, understood the medications' dosages:**				
Yes	1,086 (70.7)	490 (70.7)	596 (70.6)	0.999
No	451 (29.3)	203 (29.3)	248 (29.4)	
*Total*	*1,537 (100.0)*	*693 (100.0)*	*844 (100.0)*	
**Usually, understood the reasons for medications' prescription:**				
Yes	1,041 (68.0)	487 (70.3)	554 (66.0)	0.079
No	491 (32.0)	206 (29.7)	285 (34.0)	
*Total*	*1,532 (100.0)*	*693 (100.0)*	*839 (100.0)*	
**Usually, understood the hours of medications' intake:**				
Yes	1,213 (79.2)	543 (78.2)	670 (80.0)	0.411
No	318 (20.8)	151 (21.8)	167 (20.0)	
*Total*	*1,531 (100.0)*	*694 (100.0)*	*837 (100.0)*	
**Usually, understood the duration of medications' intake:**				
Yes	1,179 (76.9)	532 (76.5)	647 (77.2)	0.761
No	354 (23.1)	163 (23.5)	191 (22.8)	
*Total*	*1,533 (100.0)*	*695 (100.0)*	*838 (100.0)*	
**Usually, understood the side effects of the treatment:**				
Yes	1,009 (65.9)	437 (62.9)	572 (68.5)	0.023
No	521 (34.1)	258 (37.1)	263 (31.5)	
*Total*	*1,530 (100.0)*	*695 (100.0)*	*835 (100.0)*	
**Usually, understood the cost of treatment:**				
Yes	611 (39.9)	278 (40.1)	333 (39.7)	0.917
No	920 (60.1)	415 (59.9)	505 (60.3)	
*Total*	*1,531 (100.0)*	*693 (100.0)*	*838 (100.0)*	

a *Absolute numbers and column percentages (in parentheses). Discrepancies in the totals are due to missing values for different outcome variables*.

b *P-values from Fisher's exact test*.

There were no sex-differences for the outcome variables related to the information about medication use, except the understanding of side effects of the treatment which was higher in women than in men (68 vs. 63%, respectively, *P* = 0.02) (Table 1).

In crude (unadjusted) logistic regression models (Table 2), a younger age, a lower educational attainment, urban residence, unemployment, and poverty were all associated with lack of understating of most of the outcome variables (medications' names, their dosages, reasons for intake, hours of intake, duration of intake, side effects, and treatment cost). Conversely, there were no relationships with sex (except, side effects of medications), or marital status (no significant associations at all).

**Table 2:** Socio-demographic predictors of the information about medication use provided by family physicians to primary health care users in Albania.

**Predictor**	**Crude (unadjusted) models**		**Multivariable-adjusted models**^**b**^	
	**OR (95% CI)^**a**^**	***P*^**a**^**	**OR (95% CI)**	***P***
**Usually, not understanding the medications'** ***names*** **prescribed by family physicians**				
**Sex:**				
Women	1.0 (0.8–1.3)	0.753		
Men	1.0 (reference)			
**Age-group:**		**0.004 (2)**^**c**^		**<0.001 (2)**
<40 years	1.7 (1.2–2.3)	0.001	2.3 (1.6–3.2)	<0.001
40–64.9 years	1.3 (1.0–1.7)	0.078	1.4 (1.1–1.8)	0.018
≥65 years	1.0 (reference)	–	1.0 (reference)	–
**Marital status:**				
Other	1.1 (0.8–1.5)	0.496		
Married	1.0 (reference)			
**Place of residence:**				
Urban	1.4 (1.1–1.8)	0.002	1.8 (1.4–2.3)	<0.001
Rural	1.0 (reference)		1.0 (reference)	
**Education:**		**0.011 (2)**		**<0.001 (2)**
Low	1.6 (1.2–2.2)	0.003	2.2 (1.6–3.2)	<0.001
Middle	1.4 (1.0–1.9)	0.040	1.6 (1.2–2.2)	0.004
High	1.0 (reference)	–	1.0 (reference)	–
**Employment status:**		**0.009 (2)**		
Employed	1.4 (1.0–1.8)	0.027		
Unemployed	1.5 (1.2–2.0)	0.003		
Retired	1.0 (reference)	–		
**Poverty level:**				
Poor	1.6 (1.2–2.0)	<0.001	1.4 (1.1–1.9)	0.005
Not poor	1.0 (reference)		1.0 (reference)	
**Usually, not understanding the medications'** ***dosages*** **prescribed by family physicians**				
**Sex:**				
Women	1.0 (0.8–1.3)	0.969		
Men	1.0 (reference)			
**Age-group:**		**0.229 (2)**^**c**^		
<40 years	1.3 (0.9–1.7)	0.112		
40–64.9 years	1.2 (0.9–1.5)	0.172		
≥65 years	1.0 (reference)	–		
**Marital status:**				
Other	1.0 (0.8–1.4)	0.856		
Married	1.0 (reference)			
**Place of residence:**				
Urban	1.2 (0.9–1.5)	0.193	1.4 (1.2–1.9)	0.002
Rural	1.0 (reference)		1.0 (reference)	
**Education:**		**<0.001 (2)**		**<0.001 (2)**
Low	2.2 (1.6–3.0)	<0.001	2.5 (1.7–3.5)	<0.001
Middle	1.7 (1.2–2.4)	0.001	1.8 (1.3–2.5)	0.001
High	1.0 (reference)	–	1.0 (reference)	–
**Employment status:**		**0.006 (2)**		**0.001 (2)**
Employed	1.2 (0.9–1.5)	0.288	1.6 (1.2–2.1)	0.003
Unemployed	1.5 (1.2–2.0)	0.002	1.6 (1.2–2.0)	0.002
Retired	1.0 (reference)	–	1.0 (reference)	–
**Poverty level:**			1	
Poor	1.8 (1.4–2.3)	<0.001	0.6 (1.2–2.0)	<0.001
Not poor	1.0 (reference)		1.0 (reference)	
**Usually, not understanding the** ***reasons*** **for medications prescribed by family physicians**				
**Sex:**				
Women	1.2 (1.0–1.5)	0.077		
Men	1.0 (reference)			
**Age-group:**		**0.146 (2)**^**c**^		**0.016 (2)**
<40 years	1.3 (1.0–1.8)	0.069	1.6 (1.1–2.1)	0.005
40–64.9 years	1.2 (1.0–1.6)	0.117	1.3 (1.0–1.7)	0.060
≥65 years	1.0 (reference)	–	1.0 (reference)	–
**Marital status:**				
Other	1.1 (0.8–1.4)	0.609		
Married	1.0 (reference)			
**Place of residence:**				
Urban	1.6 (1.3–2.0)	<0.001	1.9 (1.5–2.4)	<0.001
Rural	1.0 (reference)		1.0 (reference)	
**Education:**		**0.399 (2)**		**0.050 (2)**
Low	1.2 (0.9–1.6)	0.277	1.5 (1.1–2.0)	0.017
Middle	1.2 (0.9–1.6)	0.188	1.3 (1.0–1.8)	0.056
High	1.0 (reference)	–	1.0 (reference)	–
**Employment status:**		**0.084 (2)**		
Employed	1.2 (0.9–1.5)	0.238		
Unemployed	1.3 (1.0–1.8)	0.026		
Retired	1.0 (reference)	–		
**Poverty level:**				
Poor	1.4 (1.1–1.8)	0.002	1.4 (1.1–1.8)	0.007
Not poor	1.0 (reference)		1.0 (reference)	
**Usually, not understanding the** ***hours*** **of intake of the medications prescribed by family physicians**				
**Sex:**				
Women	0.9 (0.7–1.1)	0.386		
Men	1.0 (reference)			
**Age-group:**		**0.005 (2)**^**c**^		**<0.001 (2)**
<40 years	1.7 (1.2–2.4)	0.003	2.0 (1.4–2.9)	<0.001
40–64.9 years	1.5 (1.1–2.0)	0.008	1.6 (1.2–2.1)	0.004
≥65 years	1.0 (reference)	–	1.0 (reference)	–
**Marital status:**				
Other	1.1 (0.8–1.5)	0.578		
Married	1.0 (reference)			
**Place of residence:**				
Urban	0.9 (0.7–1.2)	0.546		
Rural	1.0 (reference)			
**Education:**		**0.052 (2)**		**0.006 (2)**
Low	1.5 (1.1–2.1)	0.015	1.8 (1.2–2.5)	0.001
Middle	1.3 (0.9–1.9)	0.097	1.4 (1.0–2.0)	0.063
High	1.0 (reference)	–	1.0 (reference)	–
**Employment status:**		**0.002 (2)**		
Employed	1.4 (1.0–1.9)	0.035		
Unemployed	1.7 (1.3–2.4)	<0.001		
Retired	1.0 (reference)	–		
**Poverty level:**				
Poor	1.3 (1.0–1.7)	0.060		
Not poor	1.0 (reference)			
**Usually, not understanding the** ***duration*** **of intake of the medications prescribed by family physicians**				
**Sex:**				
Women	1.0 (0.8–1.2)	0.760		
Men	1.0 (reference)			
**Age-group:**		**0.010 (2)**^**c**^		**<0.001 (2)**
<40 years	1.6 (1.2–2.3)	0.003	1.8 (1.3–2.5)	0.046
40–64.9 years	1.3 (1.0–1.8)	0.046	1.3 (1.0–1.8)	<0.001
≥65 years	1.0 (reference)	–	1.0 (reference)	–
**Marital status:**				
Other	1.2 (0.9–1.6)	0.358		
Married	1.0 (reference)			
**Place of residence:**				
Urban	1.1 (0.8–1.4)	0.583		
Rural	1.0 (reference)			
**Education:**		**0.144 (2)**		
Low	1.4 (1.0–1.9)	0.054		
Middle	1.2 (0.8–1.6)	0.330		
High	1.0 (reference)	–		
**Employment status:**		**0.007 (2)**		
Employed	1.3 (1.0–1.8)	0.047		
Unemployed	1.6 (1.2–2.1)	0.002		
Retired	1.0 (reference)	–		
**Poverty level:**				
Poor	1.6 (1.2–2.0)	<0.001	1.6 (1.3–2.1)	<0.001
Not poor	1.0 (reference)		1.0 (reference)	
**Usually, not understanding the** ***side effects*** **of the medications prescribed by family physicians**				
**Sex:**				
Women	0.8 (0.6–1.0)	0.021	1.4 (1.1–1.8)	0.002
Men	1.0 (reference)		1.0 (reference)	
**Age-group:**		**0.117 (2)**^**c**^		
<40 years	1.3 (1.0–1.8)	0.047		
40–64.9 years	1.2 (0.9–1.6)	0.126		
≥65 years	1.0 (reference)	–		
**Marital status:**				
Other	1.0 (0.8–1.3)	0.892		
Married	1.0 (reference)			
**Place of residence:**				
Urban	1.1 (0.9–1.3)	0.488	1.3 (1.0–1.7)	0.018
Rural	1.0 (reference)		1.0 (reference)	
**Education:**		**0.007 (2)**		**0.004 (2)**
Low	1.4 (1.1–1.8)	0.021	1.4 (1.0–2.0)	0.026
Middle	1.0 (0.7–1.3)	0.829	0.9 (0.7–1.3)	0.622
High	1.0 (reference)	–	1.0 (reference)	–
**Employment status:**		**0.002 (2)**		**0.001 (2)**
Employed	1.1 (0.9–1.4)	0.454	1.3 (1.0–1.7)	0.068
Unemployed	1.6 (1.2–2.0)	0.001	1.7 (1.3–2.2)	<0.001
Retired	1.0 (reference)	–	1.0 (reference)	–
**Poverty level:**				
Poor	1.4 (1.1–1.8)	0.002	1.3 (1.0–1.6)	0.038
Not poor	1.0 (reference)		1.0 (reference)	
**Usually, not understanding the** ***cost*** **of the medications prescribed by family physicians**				
**Sex:**				
Women	1.0 (0.8–1.2)	0.881		
Men	1.0 (reference)			
**Age-group:**		**0.255 (2)**^**c**^		
<40 years	1.0 (0.8–1.4)	0.829		
40–64.9 years	1.2 (1.0–1.5)	0.124		
≥65 years	1.0 (reference)	–		
**Marital status:**				
Other	0.8 (0.7–1.1)	0.203		
Married	1.0 (reference)			
**Place of residence:**				
Urban	1.0 (0.9–1.3)	0.673		
Rural	1.0 (reference)			
**Education:**		**0.096 (2)**		
Low	1.2 (0.9–1.6)	0.188		
Middle	0.9 (0.7–1.2)	0.595		
High	1.0 (reference)	–		
**Employment status:**		**0.018 (2)**		**0.034 (2)**
Employed	1.1 (0.9–1.5)	0.283	1.2 (1.0–1.6)	0.103
Unemployed	1.4 (1.1–1.9)	0.005	1.4 (1.1–1.8)	0.012
Retired	1.0 (reference)	–	1.0 (reference)	–
**Poverty level:**				
Poor	1.7 (1.4–2.2)	<0.001	1.7 (1.3–2.2)	<0.001
Not poor	1.0 (reference)		1.0 (reference)	

a *Odds ratios (OR: usually not understood vs. usually understood), 95% confidence intervals (95% CI) and p-values (P) from binary logistic regression*.

b *All variables presented in the table were included in logistic regression models in a backward stepwise elimination procedure with a p-value to exit set at >0.10. Empty cells refer to the variables excluded from the logistic models*.

c *Overall p-values and degrees of freedom (in parentheses)*.

In multivariate logistic regression models, upon adjustment for all socio-demographic factors in a backward stepwise elimination procedure, poverty remained a particularly strong and significant predictor for the lack of understanding of all outcomes (medications' names: OR = 1.4, 95% CI = 1.1–1.9; dosages: OR = 1.6, 95% CI = 1.2–2.0; reasons for intake: OR = 1.4, 95% CI = 1.1–1.8; duration of intake: OR = 1.6, 95% CI = 1.3–2.1; side effects: OR = 1.3, 95% CI = 1.0–1.6; and treatment cost: OR = 1.7, 95% CI = 1.3–2.2), except the hours of medications' intake. Likewise, lower education was another strong predictor for the lack of understanding of most of the outcomes (medications' names: OR = 2.2, 95% CI = 1.6–3.2; dosages: OR = 2.5, 95% CI = 1.7–3.5; reasons for intake: OR = 1.5, 95% CI = 1.1–2.0; hours of intake: OR = 1.8, 95% CI = 1.2–2.5; and side effects: OR = 1.4, 95% CI = 1.0–2.0), except the duration of medications' intake and the treatment cost. Furthermore, younger age remained a significant determinant of lack of understanding of medications' names (OR = 2.3, 95% CI = 1.6–3.2), reasons for intake (OR = 1.6, 95% CI = 1.1–2.1), hours of intake (OR = 2.0, 95% CI = 1.4–2.9), and duration of intake (OR = 1.8, 95% CI = 1.3–2.5). Also, urban residence remained a significant predictor of lack of understating of medications' names (OR = 1.8, 95% CI = 1.4–2.3), dosages (OR = 1.4, 95% CI = 1.2–1.9), reasons for intake (OR = 1.9, 95% CI = 1.5–2.4), and side effects of the treatment (OR = 1.3, 95% CI = 1.0–1.7) (Table 2).

## Discussion

### Summary of the Findings

The main findings of this study consist of a low level of understanding of the information about use of medications prescribed by family physicians. Thus, overall, 21–60% of participants did not understand their medications. In particular, 60% of participants usually did not understand the *cost* of the treatment, probably due to the different medication brands changing rapidly in the Albanian market. However, such a low level of understating is a cause of concern and should be further investigated.

Overall, less understanding of medication use was particularly high among the poor and those with low education and among urban residents, irrespective of socioeconomic status.

### Comparison With the Literature Reports

The lack of understanding of the information on medication use by lower socioeconomic communities, evidenced in the actual study, could be partly explained by their lower HL levels. Indeed, the international scientific research reports that poor socio-economic circumstances, poverty and low education are consistently associated with a lower HL across various geographical areas, population groups, and settings (5, 6, 16–19).

These international findings about HL determinants are similar to those reported in Albania. A population-based study conducted during 2012–2014 in Tirana, the capital of Albania, reported that mean HL scores were significantly lower among individuals aged ≥66 years, individuals with lower education, low social status, and poor economic status (20). There exists a striking concordance between the determinants of HL yielded by the HL study in Albania (20) with the determinants of understanding of medicine information provided by the family physicians that are generated by this study. In other words, the groups exhibiting significantly lower understanding of the information about use of medications (older patients, low educated, poorer patients) are also reported to exhibit significantly lower HL scores compared to their respective counterparts.

Conversely, we found that urban residents usually lacked understanding of the information about medications' use and administration compared to their rural counterparts. This could be explained by the fact that family physicians in urban health centers have less time available to deal with individual patients due to the considerable high workload and, therefore, the information is conveyed more quickly and in a shorter form, compared to physicians operating in rural areas.

When the patient does not understand one or more elements of medication information provided by his/her family physician, he/she could have a look at the accompanying medication guide or consumer information included with any prescription medication, in order to find out more about the doubtful issue(s) that were not made sufficiently clear during the medical encounter. However, as Wolf and colleagues report (21), patients with low HL are significantly more likely than adequate HL patients to not read and/or look at the medication information included with prescription medications and to use these instructions (22). This means that more disadvantaged socioeconomic groups, generally characterized by lower HL, cannot take advantage of the means intended to facilitate patients' use of medication information, thus further deepening their unfavorable position compared to higher HL patients. The international literature suggests that HL is a mediator in the relationship between education and health (23), and we can hypothesize that HL could also be a mediator between education and medication use information [this assumption needs to be tested in future studies, though]. Furthermore, HL plays a larger role among individuals with lower education than among those with higher education, but the association is not linear (19). Appropriately understanding of the medication information is associated with better self-efficacy in following the respective treatment regimens as well (24–26).

The way information is conveyed to the patients by health care staff is also important. Physicians, due to their high education and training and the technical terms included in their professional vocabulary, might unintentionally convey health and medicine use information at a higher HL level that the patient can absorb (23). In this context, even patients with adequate HL might find it difficult to understand what their physicians are conveying to them (27). A number of measures can be taken in order to ensure that patients understand what their physicians are saying to them, including open communication, communicating in a simple and understandable language, using only key points, avoiding excessive information and medical jargon, and talking slowly, using comparative examples, encouraging the patients to ask questions, and the like (27). On the other hand, other general measures can be taken on a more broad level to improve the HL of the population, through government actions, an approach which could be attractive to policy-makers as a way to ensure greater participation of public and patients in health decision-making and/or to shift the burden of improving and protecting health from the government to the individual (28). However, many of the actual interventions in this regard are not properly enabling the HL concept to guide and shape the methodologies and health education and communication being employed actually; in addition the evidence-based implementation of national policies and programs and tools needed by practitioners are not emerging with the appropriate needed pace (28).

### Study Limitations

The current study may have some limitations including the possibility of selection bias, information bias and the study design employed. This study included an almost representative sample of the adult population attending PHC services in Albania. Yet, non-users may exhibit different socio-demographic characteristics compared with users of PHC services. Hence, findings from this study should be restricted only to the adult PHC users in Albania. In addition, the instruments of data collection consisted of a structured interviewer-administered questionnaire. All the interviewers were trained and the questionnaire was initially pretested. Thus, there is no evidence of any types of information biases. However, differential reporting between various socio-demographic groups of study participants cannot be completely excluded. Furthermore, associations pertinent to cross-sectional studies should be interpreted with caution, as they are not assumed to be causal.

## Conclusion

In conclusion, regardless of the aforementioned potential limitations, this study provides important evidence on the level and socio-demographic determinants of understanding of the information about medication use among adult PHC users in a transitional former communist country in South Eastern Europe. Policymakers should be aware of the joint role and interplay between health literacy (demand side) and information provision (supply side), which both significantly influence the understanding of medication use by the general population.

## Data Availability Statement

The datasets generated for this study are available on request to the corresponding author.

## Ethics Statement

The studies involving human participants were reviewed and approved by Albanian Committee of Bio-Medical Ethics. The patients/participants provided their consent to participate in this study.

## Author Contributions

DR, GB, and PS-B contributed to the study conceptualization and design, data analysis and interpretation of the data, and writing of the article. ET, SI, AY, and HB commented extensively on the manuscript. All authors have read and approved the submitted manuscript.

## Conflict of Interest

The authors declare that the research was conducted in the absence of any commercial or financial relationships that could be construed as a potential conflict of interest. The handling Editor declared a past co-authorship with one of the authors GB.
